# Supplementary material for: Merkel Cell Carcinoma Display PIEZO2 Immunoreactivity
Source: J Pers Med. 2022 May 28;12(6):894. doi: 10.3390/jpm12060894 (PMC9224776; doi:10.3390/jpm12060894)
Supplement: Supplementary file 1 [file jpm-12-00894-s001.zip › jpm-1727616-supplementary.pdf]

Journal of
Personalized
Medicine

MDPI

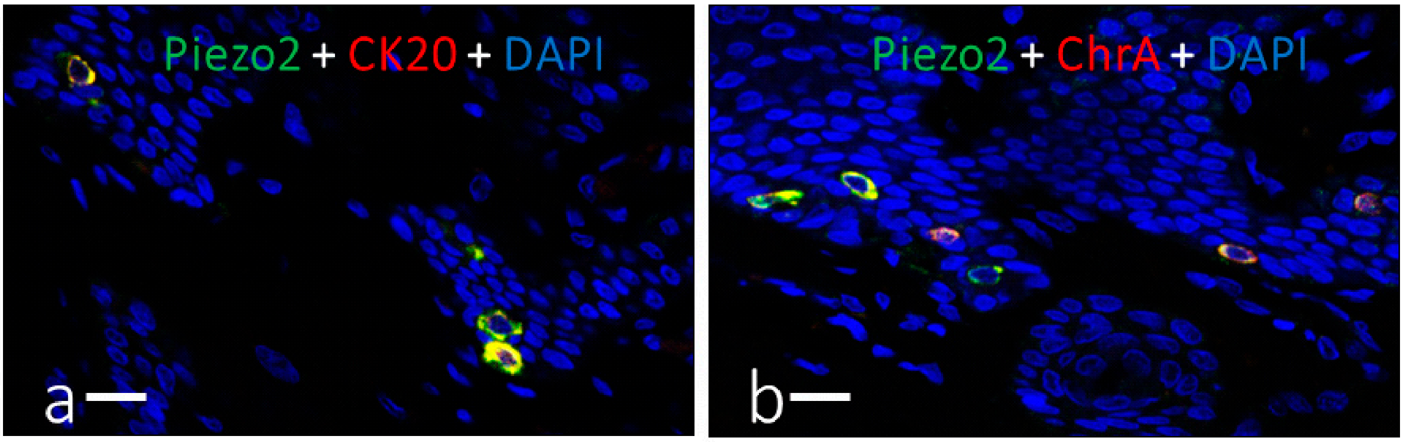

Figure S1. Epithelial CK20 Merkel cells in the epidermal basal layer demonstrate PIEZO2 immunostaining and CK20 coexpression (a,b). Scale bar 20 μm
